# Supplementary material for: The causal relationship between the human gut microbiota and pyogenic arthritis: a Mendelian randomization study
Source: Front Cell Infect Microbiol. 2024 Nov 26;14:1452480. doi: 10.3389/fcimb.2024.1452480 (PMC11629706; doi:10.3389/fcimb.2024.1452480)
Supplement: Supplementary file 1 [file DataSheet1.zip › mendelian test/ebi-a-GCST90027641.csv_forest.pdf]

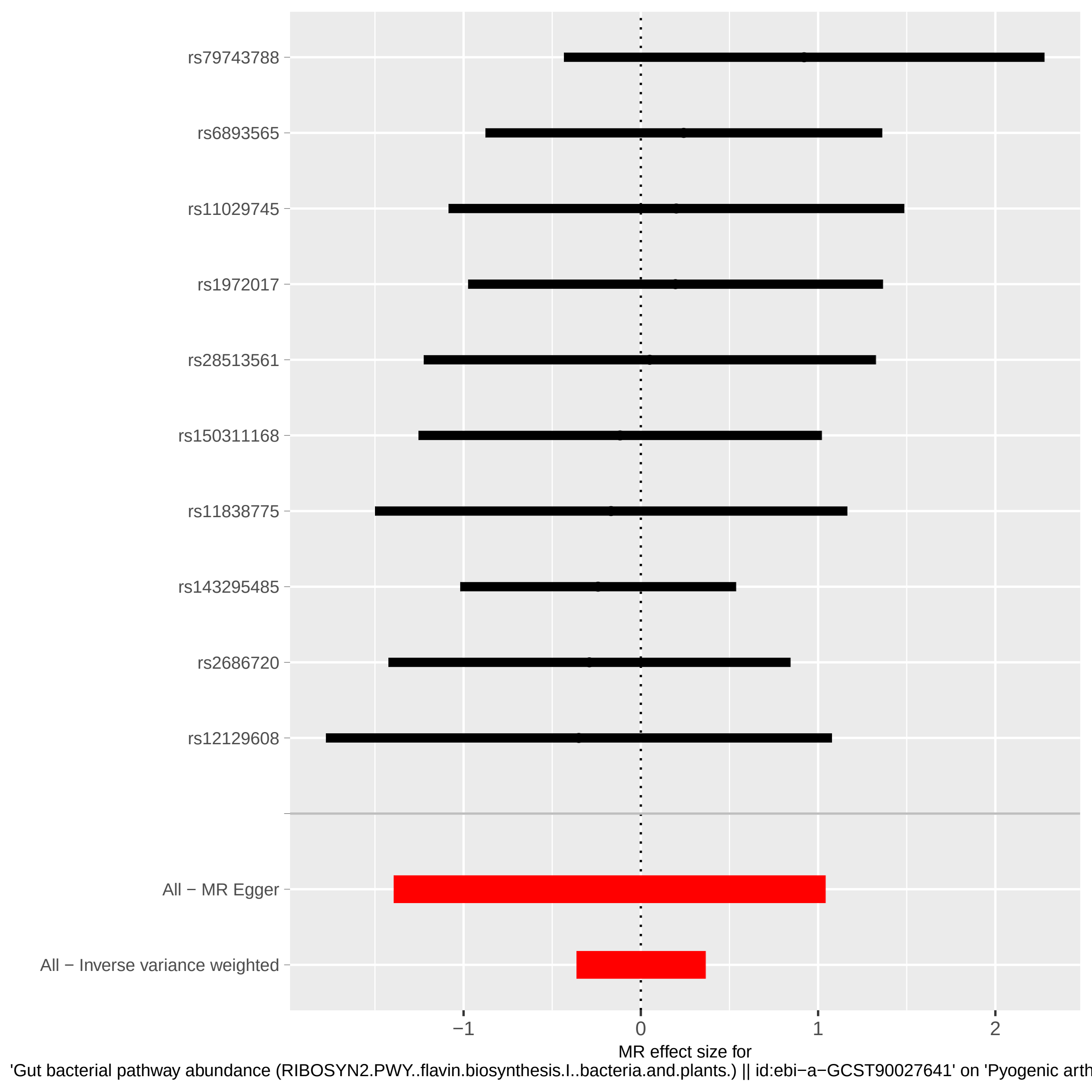

rs79743788
rs6893565
rs11029745
rs1972017
rs28513561
rs150311168
rs11838775
rs143295485
rs2686720
rs12129608
All − MR Egger
All − Inverse variance weighted
−1
0
1
2
MR effect size for
'Gut bacterial pathway abundance (RIBOSYN2.PWY..flavin.biosynthesis.I..bacteria.and.plants.) || id:ebi−a−GCST90027641' on 'Pyogenic arth
